# Supplementary material for: Celecoxib Prevents Doxorubicin-Induced Multidrug Resistance in Canine and Mouse Lymphoma Cell Lines
Source: Cancers (Basel). 2020 Apr 29;12(5):1117. doi: 10.3390/cancers12051117 (PMC7280963; doi:10.3390/cancers12051117)
Supplement: Supplementary file 1 [file cancers-12-01117-s001.pdf]

Supplementary Materials

Celecoxib Prevents Doxorubicin-Induced Multidrug Resistance in Canine and Mouse Lymphoma Cell Lines

Edina Karai, Kornélia Szebényi, Tímea Windt, Sára Fehér, Eszter Szendi, Valéria Dékay, Péter Vajdovich, Gergely Szakács and András Füredi

Table S1. Immunohistochemical findings of the two patients.

Patient 1	Markers	Results
	CD79a	Diffuse positivity.
Immunohistochemistry	CD3	Tumor cells do not express the antigen.
	Ki67	Average 30-40% positivity.
Diagnosis		Large cell immunoblastic lymphoma
Patient 2		
	CD79a	Diffuse positivity.
Immunohistochemistry	CD3	Expression cells are scattered in the tumor area.
	Ki67	Average 60% positivity.
Diagnosis		Diffuse large B-cell lymphoma

Two canine patients were diagnosed with B-cell lymphoma according to the immunohistochemistry.

Table S2. Detailed description of the used treatment protocols.

**Patient 1 modified CHOP**			
week 1	vincristine, prednisolone	week 13	no treatment (low WBC)
week 2	vincristine, cyclophosphamide, prednisolone	week 14	vincristine
week 3	vincristine, prednisolone	week 15	vincristine, prednisolone
week 4	vincristine, prednisolone	week 16	doxorubicin, prednisolone
week 5	vincristine, cyclophosphamide, prednisolone	week 17	vincristine, prednisolone
week 6	no treatment (low WBC)	week 18	vincristine, prednisolone
week 7	vincristine, prednisolone	week 19	vincristine, cyclophosphamide
week 8	vincristine	week 20	no treatment (low WBC)
week 9	vincristine, cyclophosphamide	week 21	vincristine
week 10	vincristine	week 22	vincristine
week 11	vincristine	week 23	vincristine, cyclophosphamide
week 12	vincristine, cyclophosphamide		
**Patient 2 modified CHOP**			
week 1	vincristine, prednisolone	week 17	no treatment (7 days after DOX)
week 2	prednisolone	week 18	no treatment (14 days after DOX)
week 3	prednisolone	week 19	prednisolone (21 days after DOX)
week 4	prednisolone	week 20	doxorubicin, prednisolone
week 5	cyclophosphamide	week 21	Drug Holiday
week 6	no treatment (low WBC)	week 22	Drug Holiday
week 7	vincristine, prednisolone	week 23	Drug Holiday
week 8	doxorubicin, prednisolone	week 24	Drug Holiday
week 9	no treatment (7 days after DOX)	week 25	vincristine, cyclophosphamide, prednisolone
week 10	no treatment (14 days after DOX)	week 26	vincristine, prednisolone
week 11	no treatment (21 days after DOX)	week 27	prednisolone
week 12	doxorubicin	week 28	prednisolone
week 13	no treatment (7 days after DOX)	week 29	prednisolone
week 14	no treatment (14 days after DOX)	week 30	prednisolone

week 15	no treatment (21 days after DOX)	week 31	prednisolone
week 16	doxorubicin	week 32	prednisolone

Two canine patients were treated according to the modified CHOP protocol. In case of Patient 2. between two doxorubicin treatment the bone marrow recovery time was 28 days.

Table S3. Cell surface markers used for immunophenotyping CLBL-1 cells.

CD markers			
		CD14	-
CD45	+	CD3	-
MHC II	+	CD4	-
CD79αcy	+	CD5	-
		CD21	-
		CD11d	-

The immunophenotype of CLBL-1 cells were verified according to (Rütgen et al., 2010. Leukemia Research) [1].

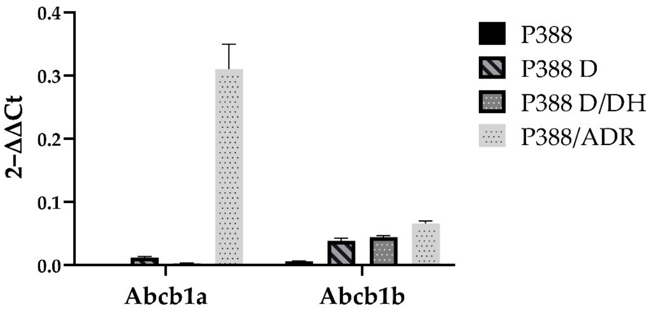

Figure S1. mRNA expression of P388 cells after DOX treatment and following a drug holiday including data obtained from P388/ADR cells (doxorubicin selected subline of P388).

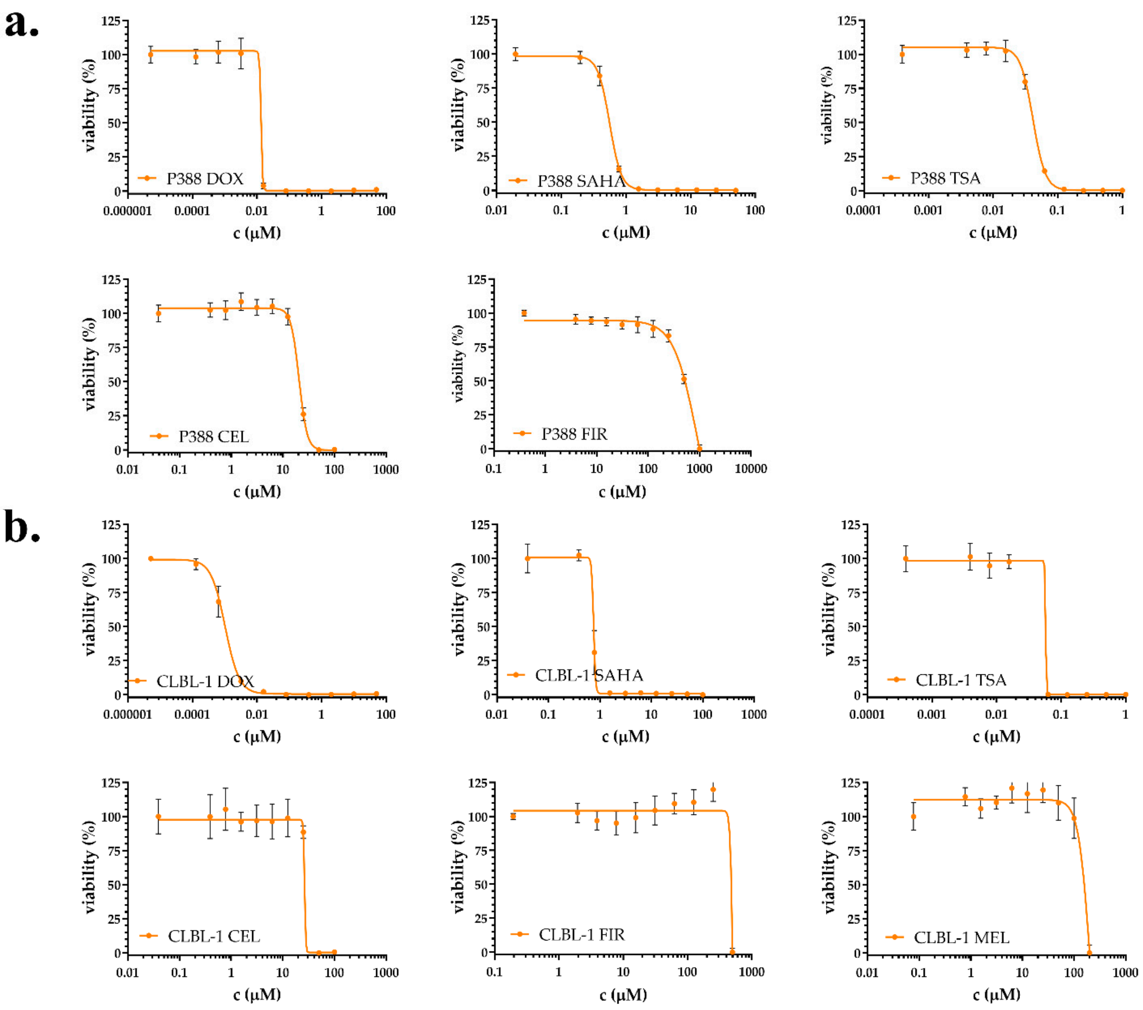

Figure S2. Citotoxicity curves of P388 (**a**) and CLBL-1 (**b**) cells showing drug sensitivity to Doxorubicin (DOX), SAHA, Trichostatin A (TSA), Celecoxib (CEL) and Firocoxib (FIR) for both cell lines. Additionally, Meloxicam (MEL) was tested only on CLBL-1 cells (**b**).

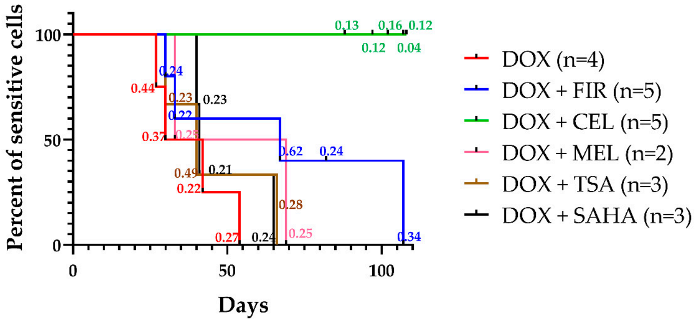

Figure S3. Kaplan-Meier curves of cell cultures including actual MAF values. Cells were considered resistant at MAF ≥ 0.2. DOX (red) in combination with FIR (blue), CEL (green), MEL (pink), TSA (brown) and SAHA (black) were tested in 9 sequential treatment cycles.

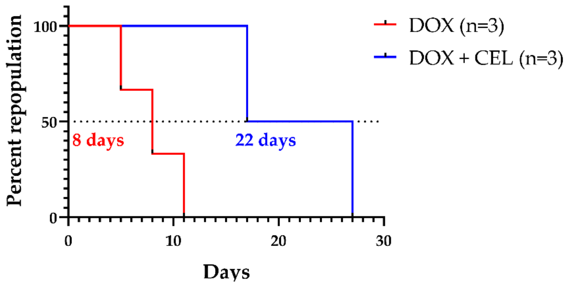

Figure S4. Kaplan-Meier curves of cell cultures with including days of repopulation time. CEL+DOX in combination increase repopulation time in both cell lines compared to DOX treatment (22 vs 8 days, respectively).

Supplementary Materials 1. Cytology Reports

Centroblastic type (Kiel) monomorphic subtype composed of more than 60% centroblasts, which are large cells with scant basophilic cytoplasm, a round nucleus, fine chromatin pattern, and 2–4 basophilic prominent nucleoli located in the margin [2]. They are 10–30 μm in diameter, and the nucleus is less completely heterochromatic than that of in small lymphocyte (they sometimes referred to as large lymphocytes or lymphoblasts) [3]. The cytoplasm is pale blue and more abundant than in small lymphocytes. Their nuclei are 1.5-3 times the size of a red blood cells (RBC) or larger to up to 4 times the size of an RBC. Some nuclei have one moderately large incision (or cleaved). The nuclei of them have a fine diffuse and light chromatin pattern. Nucleoli are prominent, and can be well visible with characteristic margins, and even multiple and/or prominent. The cytoplasm is abundant and often basophilic and may completely encircle the nucleus. Occasionally pale Golgi zone is seen besides the nucleus, at the incision of it. Mitotic figures were estimated by looking at 5 cellular fields under 40x power. In this case it was moderate: 2-3 mitotic figures [4].

Supplementary Methods 1. Method of Cytology Sampling

Criteria for the involvement of bone marrow by lymphoma were: (1) the presence of > 20% lymphocytes in the sample and/or (2) the presence of large/atypical lymphocytes, even if the proportion was lower than 20% of all nucleated cells (ANC) [5].

The lymph node and bone marrow samples were taken under general anaesthesia. The dogs were anaesthetized (propofol /AstraZeneca Co., Cambridge, UK/ 5 mg/BW kg iv, isoflurane/Abbott Ltd., Budapest, Hungary/ 1.5–2.5 V/V%, fentanyl/Gedeon Richter Plc., Budapest, Hungary/constant rate infusion 0.01 to 0.04 mg/BWkg/hr) and an enlarged lymph node was excised for routine histological and immunohistochemical examination. Bone marrow aspirates were taken for cytological analysis by using a Jamshidi needle from the iliac crest (crista iliaca externa). The aspirates were smeared and stained with a staining kit (Quick panoptic staining kit: Reagens Ltd., Budapest, Hungary) for cytological evaluation.

Sternal recumbency was used for the wing of the ilium Once the patient was sedated with the above protocol, a BMA was performed using a standard technique [6]. A 2.5cm × 2.5cm area of the skin was shaved, cleansed, and disinfected with chlorhexidine. Once the site was prepped and local anaesthesia (1–2 mL of lidocaine 2%) injected into the skin, subcutaneous tissues and on the periosteum of the bone, a small nick was made in the skin with a sterile #11 scalpel blade. The BM needle was angled slightly medially and parallel to the wing of the lium. A 15-gauge BM needle (with the stylet locked in place) was then firmly pushed through the subcutaneous tissues and the dense outer layer of the bone into the marrow. cavity. Once the needle was in contact with the surface of the bone, it was rotated into the bone in a clockwise/counter-clockwise motion. The stylet was then removed, and a 10–12 mL syringe containing 50 μL of 10% EDTA was attached to the needle, and vigorous suction was applied to withdraw a small amount of liquid marrow material into the syringe. The needle and syringe were then removed en bloc from the patient through application of firm traction to the needle. Immediately after collection of the sample, blood-contaminated BM was directly applied to glass slides (direct smears). A second slide was placed on top of the sample to

spread the material, and was then gently pulled to the end of the first slide to create a smear. A minimum of five slides were made for each BMA site.

References

1. Rütgen, B. C.; Hammer, S. E.; Gerner, W.; Christian, M.; de Arespacochaga, A. G.; Willmann, M.; Kleiter, M.; Schwendenwein, I.; Saalmüller, A. Establishment and Characterization of a Novel Canine B-Cell Line Derived from a Spontaneously Occurring Diffuse Large Cell Lymphoma. *Leuk. Res.*, **2010**, *34*, 932–938. https://doi.org/10.1016/j.leukres.2010.01.021.
2. Fournel-Fleury, C.; Magnol, J. P.; Bricaire, P.; Marchal, T.; Chabanne, L.; Delverdier, A.; Bryon, P. A.; Felman, P. Cytohistological and Immunological Classification of Canine Malignant Lymphomas: Comparison with Human Non-Hodgkin's Lymphomas. *J. Comp. Pathol.*, **1997**, *117*, 35–59.
3. Raskin, R.; Meyer, D. Canine and Feline Cytology-2nd Edition https://www.elsevier.com/books/canine-and-feline-cytology/9781416049852 (accessed 2 Mar 2020).
4. Sözmen, M.; Tasca, S.; Carli, E.; De Lorenzi, D.; Furlanello, T.; Caldin, M. Use of Fine Needle Aspirates and Flow Cytometry for the Diagnosis, Classification, and Immunophenotyping of Canine Lymphomas. *J. Vet. Diagn. Invest.*, **2005**, *17*, 323–330. https://doi.org/10.1177/104063870501700404.
5. Aubry, O. A.; Spangler, E. A.; Schleis, S. E.; Smith, A. N. Evaluation of Bone Marrow Aspirates from Multiple Sites for Staging of Canine Lymphoma and Mast Cell Tumours. *Vet. Comp. Oncol.* **2014**, *12*, 58–66. https://doi.org/10.1111/j.1476-5829.2012.00331.x.
6. Townsend, F. I. Bone Marrow Aspiration in Dogs and Cats. *Lab. Anim. (NY)*, **2008**, *37*, 497–498. https://doi.org/10.1038/laban1108-497.

© 2020 by the authors. Licensee MDPI, Basel, Switzerland. This article is an open access article distributed under the terms and conditions of the Creative Commons Attribution (CC BY) license (http://creativecommons.org/licenses/by/4.0/).
